# Special Issue: “Role of Extracellular Vesicles in Immunology”

**DOI:** 10.3390/ijms26136479

**Published:** 2025-07-05

**Authors:** Valeria Longo, Paolo Colombo

**Affiliations:** Institute for Biomedical Research and Innovation, National Research Council of Italy (IRIB-CNR), 90146 Palermo, Italy

Cell-to-cell communication is fundamental to many physiological processes and can occur through soluble factors or direct contact [1]. Recently, increasing evidence has highlighted the crucial role of extracellular vesicles (EVs)—including exosomes, microvesicles, and apoptotic bodies—as a novel mode of intercellular communication [2,3,4,5]. These lipid bilayer-bound particles are capable of carrying a diverse array of bioactive molecules such as cytokines, microRNAs, lipids, etc., making them key players in the coordination of innate immunity [3,6,7]. EVs are highly heterogeneous in composition, origin, and function, reflecting the state and environment of their donor cells [4,8]. This diversity underlines their involvement in various cellular functions, from gene regulation to metabolism, and their emerging significance in human toxicology and disease diagnostics [9]. To standardize the nomenclature, the International Society for Extracellular Vesicles has developed the Minimal Information for Studies of Extracellular Vesicles (MISEV) [10,11]. Furthermore, EVs are emerging as promising drug delivery systems due to their natural biogenesis, biocompatibility, and ability to overcome biological barriers [12]. Therefore, studies on EV isolation, biochemical characterization, and advancements in biomarker discovery and therapeutics are of paramount importance for the development of strategic target EVs in several medical applications [13,14]. This Special Issue, entitled “Role of Extracellular Vesicles in Immunology”, includes original and review articles shedding some light on the capability of EVs to modulate immune responses and dealing with their biochemical characterization.

As such, we can divide this Special Issue into different sections depending on the topics covered in the articles.

**Advancements in Biomarker Discovery and Therapeutics**

First of all, Aloi et al. provided a comprehensive overview of the role of immune-related extracellular vesicles, describing in vitro and in vivo studies aimed at elucidating the mechanisms by which EVs modulate human immunity and addressing the intricate interplay between extracellular vesicles and immunity. Indeed, the authors provide data about the active role of EVs in the modulation of maternal immune response, highlighting their role during pregnancy. Overall, this paper described the advantages and therapeutic potential of extracellular vesicles from different immune cells to various immunological disorders, including autoimmune diseases, infectious diseases, and cancer [15].

Salvat-Rovira et al. evaluated the use of ExoGAG for isolating EVs from cerebrospinal fluid as a source of biomarkers for central nervous system diseases. ExoGAG was optimized and compared to ultracentrifugation, showing that ExoGAG yields higher EV concentrations and comparable levels of specific microRNAs (miRNAs) while minimizing contamination from intracellular components. The method is simpler, requires less specialized equipment, and is suitable for clinical settings, demonstrating that ExoGAG has potential for biomarker discovery and analysis in central nervous system (CNS) diseases, offering a clinically compatible alternative to ultracentrifugation. Focusing on the same topic, Testa at al. explored how hypoxic human microglia promote angiogenesis through extracellular vesicle release. Using in vitro and in vivo models, their study demonstrates that EVs released under hypoxia enhance endothelial migration, tube formation, and vascular remodeling, highlighting the therapeutic potential of microglial EVs in stroke recovery and providing insights into microglia–endothelium interactions in ischemic conditions. The authors demonstrate that EVs may serve as promising biomarkers for chronic inflammatory diseases by reflecting disease-specific molecular signatures and mediating immune responses [16]. Similarly, Vázquez-Mera provides an in-depth analysis of the proteomic profiles of small extracellular vesicles derived from granulocytes (eosinophils and neutrophils) and CD4+ T-helper (TH) cell subsets (TH1, TH2, and TH17). This study employs both qualitative (DDA) and quantitative (DIA-SWATH) proteomic approaches to identify distinct protein signatures in sEVs, highlighting their potential as biomarkers for chronic inflammatory diseases such as asthma and COPD [17].

**Understanding EV-Induced modulation**

Many papers have shown that EV cargo is able to modulate bystander cells by transferring bioactive molecules that alter their function, phenotype, and inflammatory responses [18,19]. Lasser et al. explored the role of microRNA-125a-5p (miR-125a-5p) in the generation of myeloid-derived suppressor cells (MDSCs) in melanoma, focusing on its mechanisms and implications for immunosuppression. The study demonstrates that miR-125a-5p, enriched in tumor-infiltrating MDSCs and melanoma EVs, plays a critical role in modulating myeloid cell activation via an NF-κB-dependent pathway. Using the RET transgenic melanoma mouse model, the authors showed that miR-125a-5p correlates with tumor progression and induces MDSC-like characteristics, such as increased PD-L1 expression, IL-6 secretion, and reactive oxygen species (ROS) production. This study provides valuable insights into how melanoma-derived EVs reprogram myeloid cells to suppress anti-tumor immunity, emphasizing the role of miR-125a-5p as a key regulator. The findings could suggest strategies to counteract MDSC-mediated immunosuppression and improve melanoma immunotherapy outcomes [20]. Furthermore, Cardoso Garcia et al. investigated the impact of extracellular vesicle-depleted serum on human brain microvascular endothelial cells over different time intervals (2 and 24 h), providing a comprehensive analysis of cellular responses, extracellular vesicle characteristics, and their biological activity, offering relevant insights into EV-mediated cellular communication [21].

**Molecular Approaches mediated by extracellular vesicles**

Pathogenic bacteria employ multiple strategies to evade the host immune response and establish effective infections [22]. Among these, bacterial EVs play a crucial role as mediators of intercellular communication, capable of interacting both with other bacteria and host cells [23]. As such, the classification and characterization of bacterial EVs, including their general properties, roles in infection, and effects on the host immune system, are of significant interest [24,25]. In this Special Issue, Peregrino et al. examined bacterial EVs enriched with pathogen-associated molecular patterns (PAMPs) that trigger innate immune receptors, leading to cytokine production and inflammation. These EVs also carry antigens that can activate B and T cell responses. A deeper understanding of how bacterial EVs modulate the host immune response can provide valuable insights into the pathogenesis of clinically relevant infections and support the development of EV-based diagnostic and therapeutic approaches [26].

Catalan et al. investigated how oral pathobionts are essential to inducing local inflammation within the oral cavity, contributing to the pathogenesis of diseases in the gastrointestinal tract and other distant organs [27]. The authors elucidated the principal components of oral pathobiont-derived outer membrane vesicles (OMVs) implicated in disease pathogenesis within the oral–gut axis, detailing virulence factors that OMVs carry and their interactions with host epithelial and immune cells, both in vitro and in vivo [28].

Finally, D’avila et al. focus on the role that the modulation of lipid metabolism and EVs plays in the mechanism of immune system evasion during SARS-CoV-2 infection and explore the therapeutic potential of EVs and applications for delivering therapeutic substances to mitigate viral infections [29].

Taken together, the manuscripts featured in this Special Issue highlight the interplay between biochemical and molecular research and their potential clinical applications. This convergence not only deepens our understanding of fundamental biological processes but also paves the way for the development of innovative, personalized, and precise medical interventions [30,31]. As a result, we can anticipate a future where cutting-edge biotechnological products become an integral part of everyday medical practice [32].
